# Facultative parthenogenesis in the Ryukyu drywood termite *Neotermes koshunensis*

**DOI:** 10.1038/srep30712

**Published:** 2016-07-28

**Authors:** Kazuya Kobayashi, Yasushi Miyaguni

**Affiliations:** 1 Laboratory of Insect Ecology, Graduate School of Agriculture, Kyoto University, Oiwake-cho, Kitashirakawa, Sakyo-ku, Kyoto 606-8502, Japan

## Abstract

Parthenogenesis is a relatively rare reproductive mode in nature compared to sex. In social insects, the evolution of parthenogenesis has a notable impact on their life histories. Some termites with parthenogenetic ability produce numerous non-dispersing supplementary queens asexually, whereas other castes are produced via sexual reproduction. This asexual queen succession (AQS) system is adaptive because hundreds of the asexual queens improve the reproductive potential of the colony and maintain the genetic diversity within the colony. However, the evolutionary process of the AQS system remains unclear because parthenogenetic species without this system are unknown. Here, we report facultative parthenogenesis in the drywood termite *Neotermes koshunensis*. Although the eggs produced by females isolated from males hatched, the hatching rate of those eggs was lower than that of the eggs produced by females kept with males. These parthenogenetic offspring inherited only the maternal alleles and showed high homozygosity, which indicates that the mechanism of ploidy restoration is terminal fusion. A previous study showed that most colonies of this species have a single queen or orphan; thus, the AQS system has not evolved despite their parthenogenetic ability. Further investigations of *N. koshunensis* will reveal how parthenogenesis evolved and its role in the insect societies.

Sexual reproduction is ubiquitous in nature despite its significant costs, which include the production of males and the dilution of an individual’s genetic contribution to the next generation^1^,^2^. To produce genetic diversity among offspring, the payment of these costs imposed by sex is the rule. However, recent studies have revealed a loophole to this rule in some ant^3^,^4^,^5^ and termite societies^6^,^7^,^8^,^9^. These social insects conditionally use both sexual and asexual reproduction to obtain the advantages of both strategies: non-dispersing queens are produced asexually, whereas other castes (workers, soldiers and dispersing reproductives) are produced via normal sexual reproduction. This asexual queen succession (AQS) system allows queens to maximize their genetic contribution to the next generation through the asexual reproduction of next queens while maintaining the genetic diversity within a colony by the conditional use of sex for workers. In subterranean termites, genus *Reticulitermes*, the AQS was reported in three phylogenetically distinct species with parthenogenetic ability^10^, whereas parthenogenetic species without AQS have never been found. Thus, at least in this genus, the evolution of parthenogenesis always results in the evolution of the AQS system, which indicates the substantial benefits of this system. However, this condition makes it difficult to directly test the benefits of the AQS system because no species remain at intermediate stages in the transition from an ancestral primitive to a derived and sophisticated reproductive system. To investigate the evolutionary process of the AQS system, a non-AQS species is required, despite its parthenogenetic potential.

The Ryukyu dry-wood termite *Neotermes koshunensis* is a candidate parthenogenetic species without AQS because it shows a puzzling reproductive replacement system. Termite colonies normally produce secondary reproductives upon the death or senescence of the reigning reproductives^11^. However, in a study of *N. koshunensis*, the male secondary reproductive did not emerge in the colony from which the male reproductive was removed, whereas it appeared immediately when the colony became orphaned: both male and female reproductives were lacking^12^. This result indicates that female reproductives suppress the differentiation of male secondary reproductives, which raises the question of why the female rejects her mating opportunity through this suppression. One possible explanation is the parthenogenetic ability that allows them to reproduce without mating. Moreover, the AQS system appears to be absent in *N. koshunensis* because a previous field survey demonstrated their strict monogamy^13^, whereas all of the AQS-utilizing termite species produce hundreds of asexual supplementary queens to improve the reproductive potential of the colony^9^,^14^.

To test the parthenogenetic ability of *N. koshunensis*, we performed colony-founding experiments using female-female (FF) and female-male (FM) pairs, among which we used the individuals that had molted while isolated from the opposite sex. Moreover, we genotyped the offspring produced by FF pairs at seven newly developed microsatellite loci to confirm that they had inherited only the maternal alleles.

## Results

**Figure 1:**
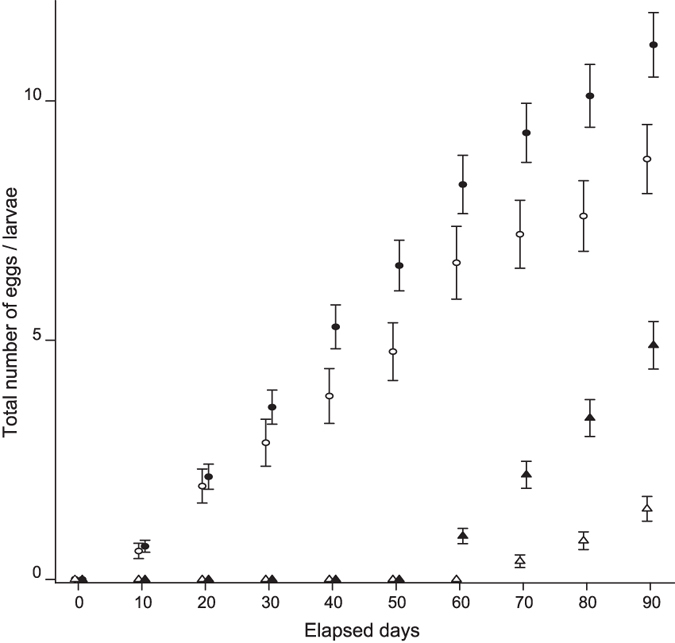
The total number of eggs and larvae produced by female-male (FM) and female-female (FF) pairs at each the observation period. Filled and open points represents mean of the data of FM and FF pairs, respectively. The shapes of the points correspond to the number of eggs (circle) and larvae (triangle). Each bar on the point indicates its standard error.

Some eggs hatched even in the FF colonies (Fig. 1). At the end of the colony-founding experiments, FM colony founders showed slightly higher survival rates compared with those from the FF colonies, although this difference was not significant (survival rate for FM: 43%, *n* = 300, and for FF: 35%, *n* = 180. Fisher’s exact test, *p* = 0.102). Focusing on the colonies where one or both founders survived to the end of the experiments, FM colonies produced more eggs and larvae than FF colonies did (for eggs, Welch’s *t*-test, *t* = −2.42, d.f. = 101.31, *p* = 0.017; for larvae, Welch’s *t*-test, *t* = −6.11, d.f. = 105.44, *p* < 0.001; Fig. 1). Although the first egg was found at the same time (10th day) for both colony types, the first hatch was observed on the 60th day for the FM colonies and on the 70th day for FF colonies. The estimated hatching rate for FM colonies was 0.88 ± 0.05 (mean ± SE, *n* = 396 eggs from 57 colonies), whereas that for FF colonies was approximately half (0.52 ± 0.07, *n* = 120 eggs from 27 colonies).

We performed a microsatellite analysis of the 5 FM and 16 FF colonies in which both founders survived to the end of the experiments, which revealed that the offspring in the FM colonies had inherited one of the paternal alleles, whereas those in the FF colonies had inherited both alleles from one of the female founders. The parthenogenetic offspring showed high homozygosity for one of the maternal alleles across all loci (Table 1). The proportions of homozygosity in the offspring for the locus that were heterozygous in the inferred mother ranged from 0.96 to 1.00 for the five genotyped loci. These proportions were significantly different from those expected under apomixes, automixis with gamete duplication, central fusion or random fusion but were consistent with the value under automixis with terminal fusion.

## Discussion

The transition stage from the ancestral to the derived trait is usually difficult to observe, especially when the evolutionary advantage is huge. Our results revealed the parthenogenetic ability of *N. koshunensis*, which remains in the intermediate stage of transition from obligately sexual to sophisticated parthenogenesis. In the species of the genus *Reticulitermes*, colony-founding experiments showed that parthenogenetic eggs of the AQS species hatched, as did the sexually produced eggs, but no larva were found from FF colonies of the non-AQS species^15^,^16^. In *N. koshunensis*, the hatching rate of parthenogenetic eggs compared to sexually produced eggs was intermediate between those of the AQS and non-AQS species in the genus *Reticulitermes*. This phenomenon in *N. koshunensis* will provide us with an opportunity to examine how the ability to reproduce parthenogenetically develops into a major feature of the breeding system of social insects as in AQS.

One of the hypothetical mechanisms countering the evolution of parthenogenesis is inbreeding depression, which reduces fitness due to the expression of recessive deleterious alleles and/or the loss of heterosis in parthenogenetic offspring^17^. This mechanism may explain the low hatching rates of parthenogenetic eggs observed in *N. koshunensis* because their ploidy level was restored by terminal fusion after meiosis (Table 1), which resulted in highly homozygous offspring. If so, recessive deleterious alleles must be purged in parthenogenetic offspring; thus, a high hatching rate is expected in parthenogenetic eggs of the parthenogens. Therefore, by using this system, we can experimentally test whether the recessive deleterious alleles and/or heterosis can be a barrier to the evolution of parthenogenesis.

Tychoparthenogenesis is presumed to be one of the intermediate stages from a sexual ancestor to parthenogenesis, which is the spontaneous hatching of a small proportion of unfertilized eggs in a normally sexually reproducing species as an error during meiotic divisions^18^,^19^. In *N. koshunensis*, the parthenogenesis hatching rate was approximately half, which is too high to be considered a mere accident in meiosis. Thus, parthenogenesis may have some functions in the life cycle of this species. A previous study showed that female reproductives suppress the differentiation of the male secondary reproductives^12^. In this study, we confirmed that the offspring in the FM colonies had inherited one of the paternal alleles, which indicated that females with a mating partner do not reproduce parthenogenetically. Integrating these results, the parthenogenetic ability may be used in the colony without male reproductives. Notably, the hatching rate of parthenogenetic eggs cancels out most of its evolutionary advantages compared with sexual reproduction, which dilutes maternal genetic contribution by half. Further studies focused on field colonies, especially on colonies without male reproductives, are needed to demonstrate the function of parthenogenesis in *N. koshunensis*.

In lower termites, parthenogenesis has been reported in at least four species: *Zootermopsis angusticollis*^20^, *Z. nevadensis*^20^, *Kalotermes flavicollis*^21^ and *Bifiditermes beesoni*^22^,^23^. However, the function of parthenogenesis in the life cycle of these species remains obscure. Accumulating knowledges of the reproductive system in these termite species will clarify how and why sexual reproduction persists in insect societies.

## Materials and Methods

### Sexual and asexual colony-founding experiments

We sampled six colonies of *N. koshunensis* (each colony labelled from A to F) nesting in dead branches of living trees in late April 2014 at Nishihara and Nakagusuku, Okinawa, Japan, and brought the nest branches to the laboratory of Kyoto University (Kyoto, Japan). Then, after cutting the nested branches into small blocks, all colony members, including reproductives, were collected as far as possible. This termite species shows the linear caste developmental pathway: pseudergates (older larvae, functional worker caste) are able to develop into alates through two stages of nymph caste (first nymphs and pre-alate nymphs)^11^,^24^. To obtain virgin alates of both sexes, the pseudergates and nymphs of each colony were separated by sex based on the morphology of abdominal sternites^25^, and maintained in a plastic box (221 × 141 × 37 mm or 270 × 190 × 51 mm) with damp chips of sliced Oregon pine wood at 27 °C in our laboratory until they molted and matured.

**Figure 2:**
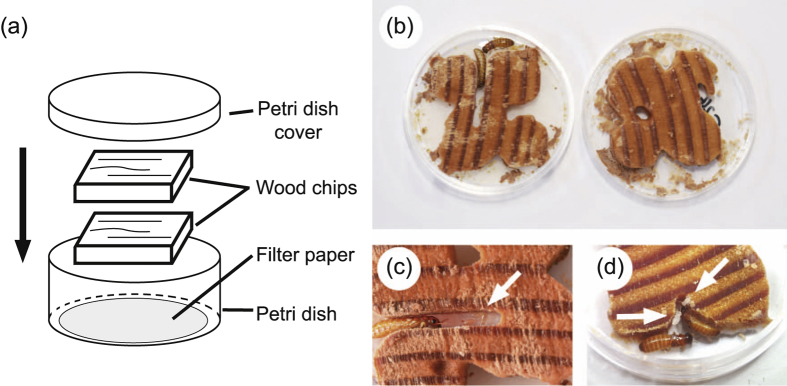
Rearing condition for the colony-founding experiments (**a**) and photographs of a typical result of the experiments (**b**–**d**). In these photographs, a female-female pair founded their colony through digging through the two chips of pine wood (**b**) and produced their eggs (indicated by the arrow) via parthenogenesis (**c**). We observed the larvae (indicated by arrows) in this experimental colony (**d**).

In early August, using the virgin female and male alates, we performed colony-founding experiments by forming sexual (female–male: FM) and asexual (female–female: FF) pairs. To obtain genetic evidence for parthenogenesis, we needed to develop genetic markers and to confirm Mendelian inheritance of these makers. For ease of confirmation, we generated founding pairs using alates originating from different colonies, i.e., the combinations of FM pairs were F_A_M_B_, F_B_M_A_, F_C_M_D_, F_E_M_F_ and F_F_M_E_, where subscripts indicate their natal colony. Moreover, to compare the reproductive rates of FM and FF pairs under the same conditions, we generated FF pairs using alates from different colonies (F_A_F_B_, F_C_F_D_ and F_E_F_F_). Each combinatorial pair was replicated 30 times and kept in 35-mm Petri dishes containing layers of a filter paper and two damp chips of Oregon pine wood at 27 °C for 90 days (22.5 × 22.5 × 4 mm for each chip; Fig. 2). Because most of the founding pairs made their nests in the upper side of the lower chip in the dishes, we opened the top wood layer, counted eggs and larvae, removed the larvae from the dish and put the top layer back in place. This method enabled us to observe the termite colonies with minimal disturbance. We performed this observation for each colony every 10 days. When the founders died, we removed their bodies from the dish at the observation period. The larvae sampled at each observation period and all individuals and eggs surviving at the end of experiments were preserved in 99.5% ethanol for subsequent genetic analysis.

Because of difficulty of tracking each egg until it hatches in the experimental colonies, we estimated the hatching rate of each colony as follows: the number of larvae hatched during a certain time period from the first hatching day was divided by the number of eggs produced during the same period from the first day of egg production. This estimate is consistent with the true hatching rate when there is no variation among the eggs in the days until hatching. To minimize the effect of variation, we decided to make the time periods for FM and FF conditions as long as possible, i.e., 40 days for FM and 30 days for FF.

### Microsatellite isolation and genotyping

We performed a microsatellite enrichment method using the magnetic beads coated by streptavidin^26^,^27^. Sixteen *N. koshunensis* colonies were sampled in August 2011 from Okinawa Island, Japan. A single worker from each colony were preserved in ethanol until DNA extraction was performed. Genomic DNA was extracted from an individual using the DNeasy tissue Kit (Qiagen). A part of the extracted DNA (20 μg) was digested by 50 units of Sau3AI (TaKaRa) at 37 °C overnight. After inactivation of the enzyme at 65 °C for 30 minutes, the mixture was purified using the QIAquick PCR purification kit (Qiagen) and eluted in 50 μl of Buffer EB (Qiagen; 10 mM Tris-Cl, pH 8.5). Then, 45 μl of the purified DNA was ligated to 1 μg of adapter (Cassette Sau3AI, TaKaRa) by 350 units of T4 DNA ligase (TOYOBO) at 16 °C overnight. After purification using the QIAquick (final elution volume was 20 μl of buffer EB), the ligated DNA was amplified by PCR. The reaction mixture consisted of 0.25 μl of ExTaq (TaKaRa), 5 μl of 10x Buffer, 4 μl of dNTP mix (10 mM each), 5 μl of cassette primer C1 (10 μM, 5′-GTACA TATTG TCGTT AGAAC GCGTA ATACG ACTCA-3′, TaKaRa), 10 μl of the purified DNA and 25.75 μl of distilled deionized H_2_O. The PCR cycling conditions were 72 °C for 5 minutes, 94 °C for 2 minutes, followed by 20 cycles of 30 seconds at 94 °C, 30 seconds at 55 °C and 2 minutes at 72 °C. After purification of the PCR product by the QIAquick (final elution volume was 50 μl of buffer EB), fragments including microsatellite regions were captured from the elution by a biotinylated oligonucleotide repeats (CA)_12_ and the magnetic beads (Dynabeads® M-280 Streptavidin; Promega) following the protocol provided by the manufacturer. The captured fragments were amplified by PCR with the above conditions, except the number of cycles was 30. The amplified DNA was cloned using pGEM®-T Easy Vector System II (Promega) and the bacterial host strain (JM109; Promega). Inserted DNA obtained from the color-positive clones were amplified by PCR. The reaction mixture consisted of 0.05 μl of ExTaq (TaKaRa), 1 μl of 10x Buffer, 0.2 μl of dNTP mix (10 mM each), 1 μl of primer mixture (1 μM each, pgF: 5′-CAGCT GGCGA AAGGG GGATG TGCT-3′ and pgR: 5′-CGTAT GTTGT GTGGA ATTGT GAGC -3′), 1 μl of the template DNA and 7 μl of distilled deionized H_2_O. The PCR cycling conditions were 94 °C 2 minutes, followed by 30 cycles of 30 seconds at 94 °C and 90 seconds at 60 °C, and one step at 72 °C for 2 minutes to complete extension at the end. The PCR products were checked for the insert size by agarose gel electrophoresis with a 100-bp ladder. The PCR products exhibiting a single band of 400–1000 bp were sequenced using a BigDye® Terminator v3.1 Cycle Sequencing Kit and 5 pmol of primer (pg01: 5′-CTCCC ATATG GTCGA CCTGC-3′ or pg02: 5′-AATTG GGCCC GACGT CGCAT-3′) on an ABI 3500 Genetic Analyzer (Applied Biosystems).

For the 18 inserts containing a microsatellite region with more than ten repeat units, primers were designed. To assess polymorphism within the Okinawa Island population, using the extracted DNA from each worker of the 16 colonies, PCRs were carried out as the following protocol. Each of the 15.2 μl reaction mixtures contained 5 pmol of each primer and 1 pmol of dyed primer (detailed information for the primers in Table S1), 0.3 μl of dNTP mix (10 mM each), 0.3 μl of 25 mM MgCl_2_, 1.5 μl of 10x Buffer, 0.1 μl of Taq DNA Polymerase (Qiagen; 1.25 U/μl) and 1 μl template DNA. The reactions were run according to the PCR cycle, which consisted of an initial denaturation step at 95 °C for 3 minutes, followed by 35 cycles of 30 seconds at 95 °C and 75 seconds at 60 °C, and one step at 72 °C for 1 minute to complete the extension at the end. The PCR products were electrophoresed with 0.5 μl of GeneScan™ 600 LIZ® dye Size Standard v2.0 on an ABI 3500 Genetic Analyzer (Applied Biosystems). Polymorphisms were found for nine loci. These alleles showed the Mendelian inheritance in the genotyping of the reproductive pairs in the FM colonies (one colony for each the combination: Table S2), and thus these loci were used in further examinations.

FF pair reproductives in which both survived to the end of experiments were genotyped at the nine loci. For the five loci where the reproductives were heterozygous, their larva and eggs were genotyped. Genomic DNA was extracted from them by a modified Chelex method. The hind legs of the reproductives, heads of the larva and the whole eggs were crushed individually in 1.5-ml microcentrifuge tubes, to which 0.6 μl of proteinase K (20 mg/ml) and 60 μl of 10% Chelex solution (10 mM Tris-HCl, 1 mM EDTA; pH 8.0) were added; the mixture was incubated at 35 °C overnight. Subsequently, the mixture was boiled at 95 °C for 15 minutes to inactivate the proteinase K. After centrifugation, the water layer was used as a template DNA. Genotyping PCR conditions were the same as those used to check the loci for polymorphisms.

## Additional Information

**How to cite this article**: Kobayashi, K. and Miyaguni, Y. Facultative parthenogenesis in the Ryukyu drywood termite *Neotermes koshunensis. Sci. Rep.* **6**, 30712; doi: 10.1038/srep30712 (2016).

## Supplementary Material

Supplementary Information

## Figures and Tables

**Table 1:** Genotypes of the offspring produced by FF pairs and expected homozygosities under the cytological mechanisms of parthenogenesis.

Locus	No. of heterozygous founders	No. of offspring		*P_*homozygosity*_*	Apomixis (*P = 0)*	Automixis			
		Homo	Hetero			Gamete duplication (*P* = 1)	Terminal fusion (*P* = 1/3–1)	Central fusion (*P* = 0–1/3)	Random fusion (*P* = 1/3)
**NK14-7**	15	51	0	1.00	***	NS	NS	***	***
**NK12-2**	19	72	0	1.00	***	NS	NS	***	***
**NK08-7**	15	74	3	0.96	***	***	NS	***	***
**NK08-2**	12	57	2	0.97	***	***	NS	***	***
**NK06-8**	7	37	0	1.00	***	NS	NS	***	***

For the number of offspring, we counted only the offspring whose mother is identified by the genotypes. *P*_*homozygosity*_*: o*bserved proportion of homozygosity in the offspring for the locus that were heterozygous in the inferred mother. *P*: expected proportion (or range of proportion) of the transition to homozygosity under a certain mechanism. Binomial tests of *P*_*homozygosity*_ under *P*: NS: not significant, ***p < 0.001. When *P* is a range, the binomial test was performed considering the *P* closest to *P*_*homozygosity*_ within the range.
