# Supplementary material for: Facultative parthenogenesis in the Ryukyu drywood termite Neotermes koshunensis
Source: Sci Rep. 2016 Jul 28;6:30712. doi: 10.1038/srep30712 (PMC4964353; doi:10.1038/srep30712)
Supplement: Supplementary Information [file srep30712-s1.pdf]

Supporting Information file

Title: Facultative parthenogenesis in the Ryukyu drywood termite *Neotermes koshunensis*

Authors: Kazuya Kobayashi[*, †], Yasushi Miyaguni[†]

Affiliations:

Laboratory of Insect Ecology, Graduate School of Agriculture, Kyoto University, Oiwake-cho, Kitashirakawa, Sakyo-ku, Kyoto 606-8502, Japan

* Correspondence to: kobakaz@kais.kyoto-u.ac.jp

† These authors contributed equally to the manuscript.

Table S1. Primer sequences and diversity statistics for nine microsatellite loci isolated from *Neotermes koshunensis.*

Locus name	Primer sequence (5'-3')	Repeat motif	Allele size range (bp)	Genetic diversity			
				N_A	H_O	H_E	*p*-value
NK14-8	F:ttggcaaccgctggagtgta<br>R:attgacctcttgcgccatgc	$(CA)_{10}$	141-169	6	0.563	0.766	0.101
*NK14-7	F:agaagctgaaaagcggtcacgg<br>R:tcatggtgactcgtgtctggtg	$(ATAG)_{10}(ACAG)_4$	208-221	5	0.313	0.387	0.108
NK14-5	F:gggaacattcgccgttgaag<br>R:cgacatttggtgcggacgta	$(GT)_{14}$	209-228	5	0.625	0.540	0.547
*NK12-2	F:tctggtcagtgacaggatagga<br>R:cccgactccaagtgtaacca	$(CA)_{15}$	321-327	2	0.125	0.121	1.000
NK12-1	F:cagatatttcatcagtatagtgggact<br>R:gcacaatacatccttaaaagttgc	$(TG)_9(TATG)_5$	150-152	2	0.000	0.226	0.029
*NK08-7	F:tttcagagccctttctgtga<br>R:gcacctggaggtttacttgg	$(AC)_{12}$	142-168	6	0.688	0.758	0.169
NK08-6	F:gcagtgagtcatgtgacggta<br>R:ggaaaaatttcatcttaggtaaacg	$(AC)_{10}$	140-156	5	0.438	0.492	0.142
*NK08-2	F:tgccaaaagtcaagggtttc<br>R:acaaggactccaaaccatgc	$(GT)_{17}$	137-156	6	0.813	0.798	0.082
*NK06-8	F:ctgcaagccggacgggtat<br>R:ccaaatctttcatatcgctatcattcg	$(CA)_{11}$	162-180	4	0.563	0.625	0.791

Shown are locus names, the forward (F) and reverse (R) primer sequence, repeat motif of the sequenced clone, allele size range in base pairs, the number of alleles (N_A), observed heterozygosity (H_O), and expected heterozygosity (H_E) in the Okinawa island (n = 16 workers, each from different colony), and *p*-value associated with departure from Hardy-Weinberg Equilibrium. Asterisks on the locus name indicate these loci showed heterozygosities in the inferred mother of the parthenogenetic offspring.

Table S2. Results of the microsatellite analysis for the five FM colonies.

Colony code	Sample name	Locus names								
		NK14-7	NK06-8	NK14-5	NK14-8	NK08-6	NK12-1	NK12-2	NK08-2	NK08-7
F_AM_B	Female founder	145/153	208/208	209/209	327/327	151/151	154/154	144/148	147/147	162/164
	Male founder	141/145	208/208	209/220	327/327	151/151	154/162	144/144	153/153	162/164
	Larva 1	145/153	208/208	209/209	327/327	151/151	154/154	144/144	147/153	162/162
	Larva 2	145/153	208/208	209/220	327/327	151/151	154/162	144/144	147/153	162/164
	Larva 3	145/153	208/208	209/220	327/327	151/151	154/162	144/148	147/153	164/164
	Larva 4	145/145	208/208	209/209	327/327	151/151	154/154	144/144	147/153	164/164
	Larva 5	145/153	208/208	209/220	327/327	151/151	154/162	144/144	147/153	162/164
	Larva 6	145/153	208/208	209/220	327/327	151/151	154/162	144/144	147/153	162/162
	Larva 7	145/145	208/208	209/209	327/327	151/151	154/154	144/144	147/153	162/164
	Larva 8	141/153	208/208	209/220	327/327	151/151	154/162	144/144	147/153	162/164
	Larva 9	145/145	208/208	209/209	327/327	151/151	154/154	144/144	147/153	162/164
	Larva 10	141/153	208/208	209/220	327/327	151/151	154/162	144/148	147/153	164/164
	Egg 1	145/153	208/208	209/220	327/327	151/151	154/162	144/148	147/153	162/162
	Egg 2	141/145	208/208	209/209	327/327	151/151	154/154	144/144	147/153	162/164
	Egg 3	141/153	208/208	209/220	327/327	151/151	154/162	144/144	147/153	162/164
	Egg 4	145/145	208/208	209/209	327/327	151/151	154/154	144/148	147/153	164/164
	Segregation ratios									
	Expected	1:1:1:1	1	1:1	1	1	1:1	1:1	1	1:2:1
	Observed	1:3:4:6	1	6:8	1	1	6:8	10:4	1	3:7:4
	p-value	0.4085	1.0	1.0	1.0	1.0	1.0	0.4401	1.0	1.0
F_BM_A	Female founder	141/145	208/208	209/209	327/327	151/151	154/154	144/148	147/153	164/164
	Male founder	141/141	208/208	209/220	327/327	151/151	154/162	144/144	147/153	162/164
	Larva 1	141/141	208/208	209/220	327/327	151/151	154/162	144/148	147/153	162/164
	Larva 2	141/145	208/208	209/220	327/327	151/151	154/162	144/144	153/153	162/164
	Larva 3	141/145	208/208	209/209	327/327	151/151	154/154	144/148	147/153	164/164
	Larva 4	141/145	208/208	209/209	327/327	151/151	154/154	144/148	147/147	164/164
	Larva 5	141/145	208/208	209/220	327/327	151/151	154/162	144/144	153/153	164/164
	Larva 6	141/141	208/208	209/220	327/327	151/151	154/162	144/148	153/153	164/164
	Larva 7	141/141	208/208	209/209	327/327	151/151	154/154	144/148	147/153	164/164
	Larva 8	141/145	208/208	209/209	327/327	151/151	154/154	144/148	147/153	164/164
	Larva 9	141/145	208/208	209/220	327/327	151/151	154/162	144/144	147/153	162/164
	Segregation ratios									
	Expected	1:1	1	1:1	1	1	1:1	1:1	1:2:1	1:1
	Observed	3:6	9	4:5	9	9	4:5	3:6	1:5:3	3:6
	p-value	0.6372	1.0	1.0	1.0	1.0	1.0	0.6372	0.6890	0.6372
F_CM_D	Female founder	141/145	208/208	209/209	327/327	151/151	154/154	144/144	147/147	164/164
	Male founder	153/153	208/208	209/225	327/327	151/151	154/162	144/144	145/147	162/162
	Larva 1	145/153	208/208	209/209	327/327	151/151	154/154	144/144	147/147	162/164
	Larva 2	145/153	208/208	209/209	327/327	151/151	154/154	144/144	147/147	162/164
	Larva 3	145/153	208/208	209/209	327/327	151/151	154/154	144/144	147/147	162/164
	Larva 4	141/153	208/208	209/209	327/327	151/151	154/154	144/144	147/147	162/164
	Larva 5	141/153	208/208	209/225	327/327	151/151	154/162	144/144	145/147	162/164
	Larva 6	141/153	208/208	209/209	327/327	151/151	154/154	144/144	147/147	162/164
	Larva 7	145/153	208/208	209/225	327/327	151/151	154/162	144/144	145/147	162/164
	Larva 8	141/153	208/208	209/225	327/327	151/151	154/162	144/144	145/147	162/164
	Larva 9	145/153	208/208	209/225	327/327	151/151	154/162	144/144	145/147	162/164
	Larva 10	141/153	208/208	209/209	327/327	151/151	154/154	144/144	147/147	162/164
	Larva 11	141/153	208/208	209/209	327/327	151/151	154/154	144/144	147/147	162/164
	Larva 12	145/153	208/208	209/225	327/327	151/151	154/162	144/144	145/147	162/164
	Larva 13	141/153	208/208	209/209	327/327	151/151	154/154	144/144	147/147	162/164
	Larva 14	145/153	208/208	209/209	327/327	151/151	154/154	144/144	147/147	162/164
	Segregation ratio									

Table S2. Continued

	Expected	1:1	1	1:1	1	1	1:1	1	1:1	1
	Observed	7:7	14	5:9	14	14	5:9	14	5:9	14
	p-value	1.0	1.0	0.7036	1.0	1.0	0.7036	1.0	0.7036	1.0
F_EM_F	Female founder	153/153	208/208	209/209	327/327	151/151	154/154	144/144	153/153	162/164
	Male founder	141/153	208/214	209/220	327/327	151/151	154/162	144/144	145/153	162/164
	Larva 1	141/153	208/214	209/220	327/327	151/151	154/162	144/144	153/153	164/164
	Larva 2	141/153	208/214	209/220	327/327	151/151	154/162	144/144	153/153	162/162
	Larva 3	141/153	208/208	209/220	327/327	151/151	154/162	144/144	153/153	162/164
	Larva 4	141/153	208/208	209/220	327/327	151/151	154/162	144/144	153/153	162/164
	Larva 5	141/153	208/208	209/209	327/327	151/151	154/154	144/144	145/153	164/164
	Larva 6	141/153	208/214	209/220	327/327	151/151	154/162	144/144	153/153	162/164
	Larva 7	141/153	208/214	209/220	327/327	151/151	154/162	144/144	153/153	162/164
	Egg 1	141/153	208/208	209/220	327/327	151/151	154/162	144/144	153/153	164/164
	Egg 2	141/153	208/214	209/209	327/327	151/151	154/154	144/144	145/153	164/164
	Egg 3	141/153	208/208	209/220	327/327	151/151	154/162	144/144	153/153	162/162
	Egg 4	141/153	208/214	209/209	327/327	151/151	154/154	144/144	145/153	162/164
	Egg 5	141/153	208/208	209/209	327/327	151/151	154/154	144/144	145/153	162/164
	Egg 6	141/153	208/214	209/209	327/327	151/151	154/154	144/144	145/153	164/164
	Segregation ratios									
	Expected	1:1	1:1	1:1	1	1	1:1	1	1:1	1:2:1
	Observed	0:13	6:7	5:8	13	13	5:8	13	5:8	2:6:5
	p-value	0.0052*	1.0	0.6951	1.0	1.0	0.6951	1.0	0.6951	0.5451
F_FM_E	Female founder	141/141	208/208	209/209	327/327	149/149	154/154	148/148	153/153	164/164
	Male founder	141/153	208/208	209/225	327/327	149/149	154/162	140/148	145/145	164/164
	Larva 1	141/141	208/208	209/225	327/327	149/149	154/162	148/148	145/153	164/164
	Larva 2	141/141	208/208	209/209	327/327	149/149	154/154	140/148	145/153	164/164
	Larva 3	141/153	208/208	209/209	327/327	149/149	154/154	148/148	145/153	164/164
	Larva 4	141/153	208/208	209/209	327/327	149/149	154/154	140/148	145/153	164/164
	Larva 5	141/153	208/208	209/209	327/327	149/149	154/154	140/148	145/153	164/164
	Larva 6	141/153	208/208	209/225	327/327	149/149	154/162	140/148	145/153	164/164
	Larva 7	141/141	208/208	209/225	327/327	149/149	154/162	148/148	145/153	164/164
	Larva 8	141/153	208/208	209/209	327/327	149/149	154/154	140/148	145/153	164/164
	Larva 9	141/153	208/208	209/225	327/327	149/149	154/162	148/148	145/153	164/164
	Larva 10	141/153	208/208	209/209	327/327	149/149	154/154	140/148	145/153	164/164
	Segregation ratios									
	Expected	1:1	1	1:1	1	1	1:1	1:1	1	1
	Observed	3:7	10	4:6	10	10	4:6	4:6	10	10
	p-value	0.6499	1.0	1.0	1.0	1.0	1.0	1.0	1.0	1.0

Colony code was consisted with the combination codes in the main text, thus the subscripts indicated natal colony of founders. Expected segregation ratios were calculated from the genotypes of parents. The *p*-values indicated the results of Fisher's exact tests. *: the *p*-values presented here were not corrected for multiple testing. As we performed the test 45 times, the value was 0.234 after the correction with the holm method.
